# A female patient carrying a novel *DMD* mutation with non-random X-chromosome inactivation from a DMD family

**DOI:** 10.1186/s12920-024-01794-x

**Published:** 2024-02-01

**Authors:** Ming-Xia Sun, Miao Jing, Ying Hua, Jian-Biao Wang, Sheng-Quan Wang, Li-Lan Chen, Liang Ju, Yan-Shan Liu

**Affiliations:** 1 Department of Neurology, Affiliated Children’s Hospital of Jiangnan University (Wuxi Children’s Hospital), Wuxi, China; 2 Department of Cardiology, Affiliated Children’s Hospital of Jiangnan University (Wuxi Children’s Hospital), Wuxi, China; 3 Department of Pediatric Laboratory, Affiliated Children’s Hospital of Jiangnan University (Wuxi Children’s Hospital), Wuxi, China

**Keywords:** DMD, Manifesting carrier, Novel mutation, X-chromosome inactivation

## Abstract

**Objective:**

To analyze the clinical phenotype and genetic characteristics of a female proband carrying a novel mutation in the *DMD* gene with non-random X-chromosome inactivation in a large pedigree with pseudohypertrophic muscular dystrophy.

**Methods:**

Clinical information of the female proband, her monozygotic twin sister, and other family members were collected. Potential pathogenic variants were detected with Multiplex Ligation-dependent Probe Amplification (MLPA) and whole-exome sequencing (WES). Methylation-sensitive restriction enzyme (HhaI) was employed for X-chromosome inactivation analysis.

**Results:**

The proband was a female over 5 years old, displayed clinical manifestations such as elevated creatine kinase (CK) levels and mild calf muscle hypertrophy. Her monozygotic twin sister exhibited normal CK levels and motor ability. Her uncle and cousin had a history of DMD. WES revealed that the proband carried a novel variant in the DMD (OMIM: 300,377) gene: NM_004006.3: c.3051_3053dup; NP_003997.2: p.Tyr1018*. In this pedigree, five out of six female members were carriers of this variant, while the cousin and uncle were hemizygous for this variant. X-chromosome inactivation analysis suggested non-random inactivation in the proband.

**Conclusion:**

The c.3051_3053dup (p.Tyr1018*) variant in the *DMD* gene is considered to be the pathogenic variant significantly associated with the clinical phenotype of the proband, her cousin, and her uncle within this family. Integrating genetic testing with clinical phenotype assessment can be a valuable tool for physicians in the diagnosis of progressive muscular dystrophies, such as Becker muscular dystrophy (BMD) and Duchenne muscular dystrophy (DMD).

**Supplementary Information:**

The online version contains supplementary material available at 10.1186/s12920-024-01794-x.

## Background

Pseudohypertrophic muscular dystrophy is an X-linked recessive genetic disorder that encompasses the milder form of Becker muscular dystrophy (BMD, MIM: 300,376) and the more severe form of Duchenne muscular dystrophy (DMD, MIM: 310,200) [1, 2]. DMD typically manifests between the ages of 3 to 5, progressing gradually and clinically characterized by calf muscle hypertrophy, a positive Gowers’ sign, significantly elevated serum creatine kinase (CK) levels, and, in advanced stages, widespread skeletal muscle atrophy. Individuals with DMD often loss the ability to walk around 10-12 years old, need assisted vertilation around 20. Most DMD patient decease between 20 and 40, primarily due to respiratory failure and end-stage heart failure (HF) [2, 3].

The genetic basis of both DMD and BMD lies in mutations within the dystrophin gene (DMD), located on chromosome Xp21.2 and consisting of 79 exons. This gene encodes dystrophin, a skeletal protein that connects cytoskeleton of muscle fibers to the extracellular matrix; therefore, dystrophin acts as stabilizer of muscle fibres during movement [4]. The spectrum of mutations within this gene is diverse, with exon deletions and duplications comprising a substantial majority, accounting for 70-80% of pathogenic mutations in DMD. These large-scale alterations in the gene structure can disrupt the normal reading frame and compromise the synthesis of functional dystrophin. Other forms of mutations including small variations, or point mutations, with nonsense mutations accounting for 13-15% of cases [5].

DMD predominantly affects males, with an incidence of approximately 3 per 10,000 live male births. While most female carriers of DMD are asymptomatic and do not show the typical signs of the disorder, a subset, referred to as manifesting carries (MCs), may experience varying degrees of muscle weakness symptoms, constituting around 2.5-19% of female carriers [6]. The severity of symptoms in MCs can vary widely. Most of the MCs show milder clinical manifestations that male patients, however, some of the affected females have similar severe symptoms as affected males. Though the reasons behind this variability are not fully understood, skewed X-chromosome inactivation (XCI) is believed to be one of the main mechanisms underlying DMD MCs [7].

In the present study, we reported a case of a female MC from a large DMD family. Whole exome sequencing (WES) identified a novel mutation in the *DMD* gene inherited within this family. XCI analysis suggested non-random inactivation in the female proband. Intriguingly, the proband has a monozygotic twin sister who has normal muscle functions despite sharing an identical genetic background. Our findings expand the spectrum of DMD mutations, and contribute valuable insights in diagnosis and monitoring strategies for female MCs.

## Methods

### Patient characteristics

The patient is a 5-year-old female who presented at the Infectious Diseases Department of Jiangnan University Affiliated Children’s Hospital in June 2023 with a complaint of “cough for 15 days with intermittent fever.” The patient has a history of muscle pain and fatigue following physical exertion. The proband has a monozygotic twin sister with normal phenotype. Upon inquiring about the family’s history, it was revealed that her cousin and uncle both exhibited varying degrees of delayed motor development and elevated CK levels. After obtaining informed consent for each participant, including the minors that were signed by their parents, clinical data and peripheral blood samples were collected from family members for this study. This research received approval from the Medical Ethics Committee of Jiangnan University Affiliated Children’s Hospital (Approval Number: [WXCH2022-07-028-2]).

### Extraction of genomic DNA

Peripheral venous blood (2 ml) was collected from family members using ethylenediaminetetraacetic acid (EDTA) as an anticoagulant.DNA extraction was performed using the Omega DNA extraction kit (Omega Corporation, USA).

### Genetic testing

Deletions and duplications in the *DMD* gene was assessed through the application of Multiplex Ligation-dependent Probe Amplification (MLPA) kit P034-A2/P035-A2 (MRC-Holland, Netherlands). Whole exome sequencing (WES) was performed for the detection of potential variant [8, 9]. Candidate variants underwent pathogenicity assessment in accordance with the guidelines established by the American College of Medical Genetics and Genomics (ACMG) [10]. Primers specific to the identified DMD gene variants were designed, and PCR amplification was carried out for variants confirmation. PCR product was sequenced using the ABI 3730XL sequencer.

### X-chromosome inactivation (XCI) testing

Genomic DNA obtained from the proband, her monozygotic twin sister and their parents was subjected to enzyme digestion using the methylation-sensitive restriction enzyme HhaI, followed by capillary electrophoresis and fragment analysis [11]. In somatic cells, the process of XCI is typically random, resulting in an approximately 50:50 ratio of cells expressing either the paternal or maternal X chromosome in female somatic cells. Any significant deviation from this 50:50 ratio suggests non-random inactivation. The evaluation of XCI varies in the literature [12, 13]. In the present study, we used an 80:20 ratio as the cutoff for identifying non-random inactivation. A ratio between 80:20 and 70:30 was considered as mildly non-random inactivation [13].

## Results

### Case presentation

The proband is a female, born as the second child of the first pregnancy and the younger monogenic twin. She was delivered via cesarean section at full term with a birth weight of 3000 g. There was no history of intrauterine or postnatal distress, and she achieved developmental milestones as follows: she could lift her head at 3 months, sit independently at 6 months, began walking at 15 months, and started saying “dada” and “mama” at around 1 year of age. Upon admission, her general examination revealed that she was alert, responsive, with no skin rashes. Palpation of her neck identified several lymph nodes approximately the size of green beans on both sides, which had a soft texture, normal mobility, and were non-tender. During lung examination, coarse breath sounds were noted, although there was no obvious wheezing or crackles. Her abdominal examination did not reveal any abnormalities. Neurological examination indicated no significant abnormalities in higher cortical functions or cranial nerves.

The proband’s highest CK value recorded was 4145 U/L (40-200U/L). In contrast, the CK value for her sister was 165 U/L. She had a history of muscle pain and fatigue following physical exertion.The proband exhibited hypertrophy in both calf muscles with a weakly positive Gowers’ sign, whereas her twin sister showed no calf muscle hypertrophy or Gowers’ sign. The 10-meter running time for the proband and her sister was 9 and 7 s, respectively. The muscle strength of the proband was assessed at level V in all her limbs. Muscle tone was within normal range. Her sensation was intact, and biceps reflexes were present in the upper limbs, while the patellar reflexes in the lower limbs were slightly weaker. There were no pathological reflexes detected. Moreover, Peabody developmental motor scales were employed to assess motor functions and skills in both the proband and her sister, revealing multiple delays in the proband (Table 1).

**Table 1:** Peabody developmental motor scales

		Proband	sister
Posture	Raw score	53	57
	Equivalent month age	55 m	67 m
Move	Raw score	170	175
	Equivalent month age	59 m	69 m
Physical Operation	Raw score	45	46
	Equivalent month age	66 m	71 m
Prehension	Raw score	50	51
	Equivalent month age	55 m	63 m
Visual Movement	Raw score	139	140
	Equivalent month age	68 m	71 m

**Fig. 1:**
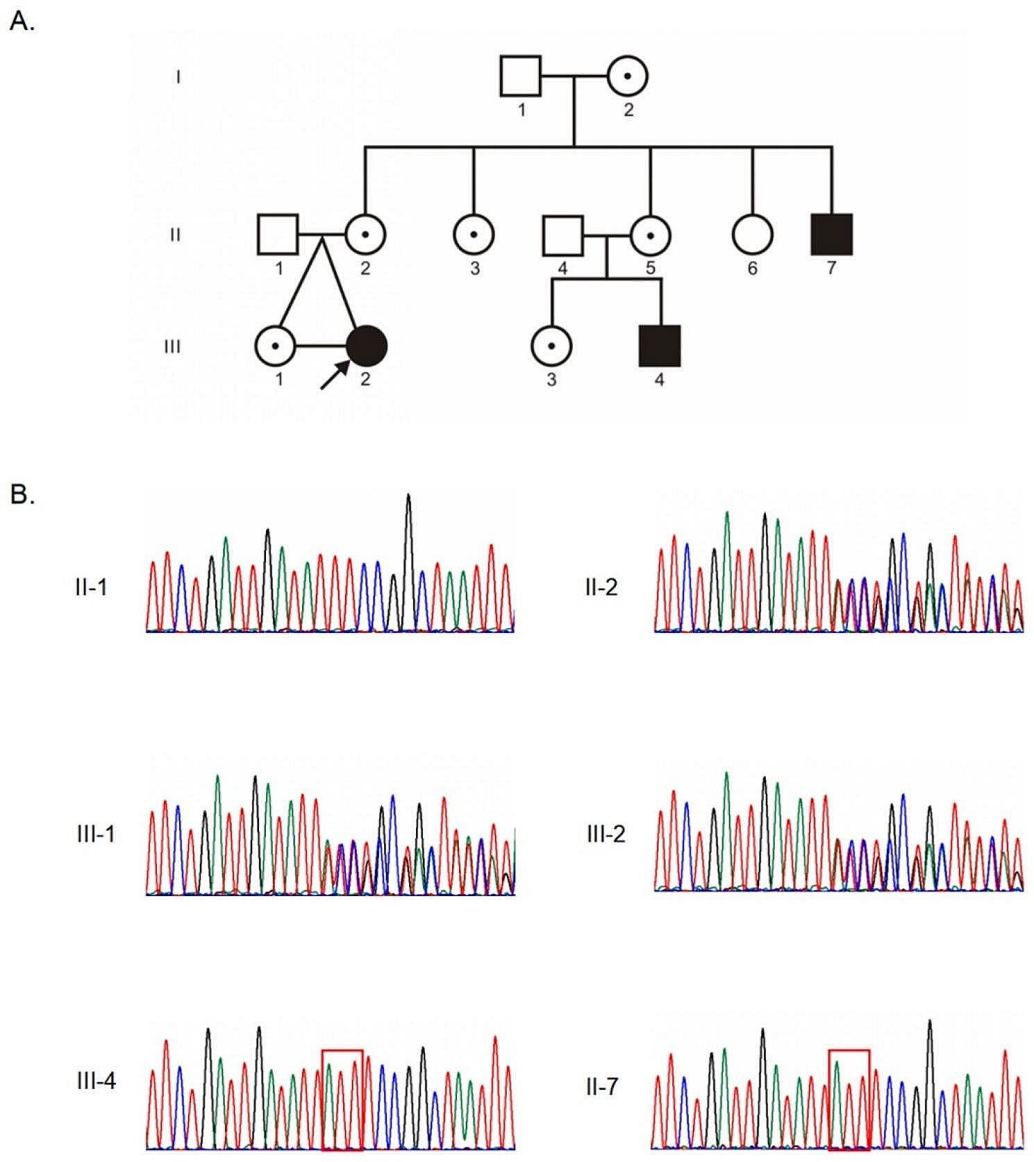
A novel mutation in the *DMD* gene is found in a big DMD family. (**A**) The pedigree chart of the family involved in the present study. Standard symbols have been used to denote sex and affected status. (**B**) Sanger sequencing confirmed the mutation in the family. The three-base-pair duplication in hemizygotes (III-4 and II-7) was indicated with rectangle

Upon further inquiry into the family’s medical history, it was revealed that her uncle and cousin had a previous diagnosis history of pseudohypertrophic muscular dystrophy (DMD) (Fig. 1A). The proband’s cousin (III-4), who is over a year old, still cannot walk independently or sit, and has elevated CK levels. The proband’s uncle (II-7) exhibited delayed motor development, lost the ability to walk and stand at around 9 years of age, and had elevated CK levels as well.

### Genetic testing

We first performed MLPA testing on the proband (III-2) and did not detect any large deletions or duplications in the *DMD* gene. Subsequently, we conducted WES, leading to the identification of a heterozygous variant in exon 23 of the *DMD* gene in the proband, NM_004006.3: c.3051_3053dup; NP_003997.2: p.Tyr1018*. This variant consisted of a three-base pair duplication that leads to a premature stop codon at position 1018 of the DMD protein, resulting in protein truncation. Further analysis involved Sanger sequencing of other family members. This revealed that six additional female family members carried this variant without any clinical symptoms related to DMD (Fig. 1B, Fig. S1). Two male family members were identified as hemizygotes, and they displayed symptoms consistent with DMD, leading to a clinical diagnosis of the condition.

**Fig. 2:**
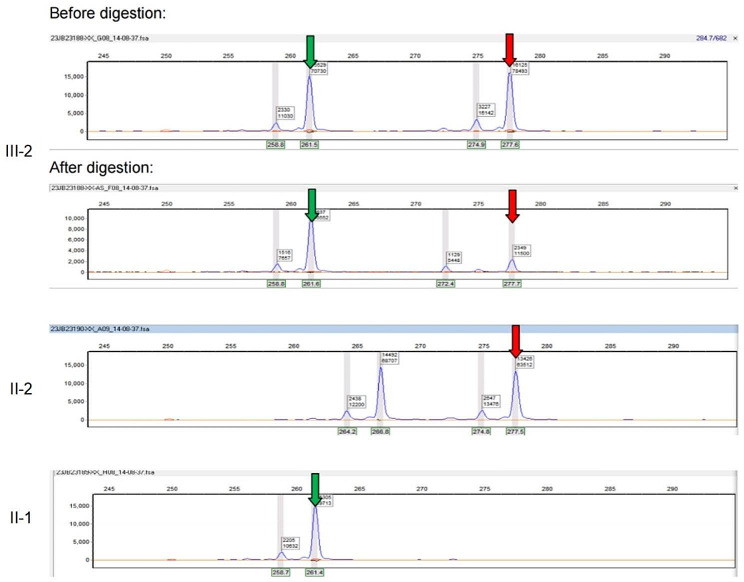
Non-random X-chromosome inactivation was identified in the proband. The green and red arrows represent the corresponding X-chromosome inherited from the proband’s father (II-1) and mother (II-2), respectively

Subsequently, we proceeded to perform X-chromosome inactivation testing on both the proband and her twin sister. The results revealed that the proband had a ratio of expressing the maternal X chromosome to the paternal X chromosome at 81:19, which strongly indicated non-random inactivation (Fig. 2). In contrast, the twin sister of the proband displayed a ratio of 76:24 for X chromosome inactivation (Fig. S2).

## Discussion

In this study, the female proband carries a heterozygous mutation in the *DMD* gene, c.3051_3053dup (p.Tyr1018*), which is located in exon 23. This three-base-pair insertion induced a nonsense mutation that resulted in the premature termination of protein translation, leading to truncation of the protein. According to the American College of Medical Genetics and Genomics guidelines from 2015 [10], this mutation is considered pathogenic.

Previous studies indicated that the age of onset in female MCs can span a wide range from 0 to 47 years [6]. Studies have found a correlation between the age of symptom onset and the severity of symptoms in female MCs. Specifically, individuals who begin to experience symptoms before the age of 10 tend to exhibit more severe and faster-progressing symptoms, akin to those observed in male DMD patients. Moreover, it has also been proposed that the severity of symptoms in female MCs might be associated with the quantity of dystrophin protein present in their muscle tissues. On the other hand. female carriers, even asymptomatic ones, may at a risk of cardiomyopathy. Around 47% of female carriers had at least one positive finding with Cardiac MR (CMR). The risk of developing cardiomyopathy increases with age [6], so cardiomyopathy is a potential problem that needs attention in female DMD variant carriers.Other study in MCs showed the incidence of cardiomyopathy was 7.3-16.7% and 0-13.3% for DMD and BMD, respectively [2]. So far, the etiology of cardiomyopathy in female carriers with DMD mutation is still under elucidated, and studies are needed to identify the best diagnostic and therapeutic strategies for affected female [14]. In the present study, in addition to genetic counseling, routine cardiovascular function tests are necessary for all carriers, including the proband.

The exact mechanisms responsible for the onset symptoms in female MCs remain incompletely understood and may involve factors such as uniparental disomy (UPD), XCI skewing (where either the maternal or paternal X chromosome has an inactivation rate exceeding 80%, allowing the X chromosome with the abnormal gene to remain active), X-autosomal translocations and Turner syndrome. Among these factors, XCI skewing has become a significant focus of current research, though the exact role of XCI skewing remains controversy [15]. It has been reported that XCI skewing is present in 33.3-100% of female MCs patients [6]. Viggiano et al. suggested some extent of correlation between the ratio of skewed XCI and severity in female MCs. Carriers with a moderate or extremely skewed XCI showed moderate/severe muscle damage, while carriers with random XCI showed mild muscle damage [14]. Nevertheless, the precise relationship between gene mutations, the pattern of XCI, and the clinical manifestations of these conditions remains unclear. Further research in this area is necessary to gain a deeper understanding of these complex interactions.

The past reports in female DMD patients in monozygotic twins suggested uneven X-chromosome inactivation was the main reason for distinct phenotypes between the twin sisters [11, 16]. An interestingly observation in this study is that proband’s monozygotic twin sister shows no related clinical manifestations. X-chromosome inactivation testing showed a slightly difference, both mild non-random inactivation in the proband (81:19) and her sister (76:24) with DNA from peripheral blood. Previous studies showed tissues derived from the same embryonic layer tend to exhibit a good correlation in X-inactivation status [14]. Given that both blood and skeleton muscle are developed from mesoderm, our results from blood may reasonably reflect the status of skeletal muscle as well. Though there is no DMD-related symptoms in the twin sister, a long-term follow-up is essential to evaluate the development of both the proband and her twin sister.

It has been reported that many MCs with DMD mutations can have elevated CK levels, marking a notable biochemical abnormality. However, the clinical manifestation of skeletal muscle symptoms, particularly severe muscle weakness, is considered a rare occurrence [17]. Apart from elevated CK levels, female DMD patients typically present with chronic progressive proximal limb muscle weakness, often accompanied by calf muscle enlargement. Notably, the symptoms observed in MCs with DMD bear a resemblance to those associated with limb-girdle muscular dystrophy, which is a genetically heterogeneous disorder and could be inherited in autosomal recessive or dominant manner [18–20]. Therefore, the similar symptoms between DMD and limb-girdle muscular dystrophy, can present a diagnostic challenge. As a result, there is a possibility that female DMD cases may be misinterpreted or overlooked, leading to delayed or inaccurate diagnoses. On the other hand, the challenge in diagnosis strengthened the importance of genetic testing in distinguishing these diseases. Moreover, even though there are a few of theraputic strategies have been approved by the U.S. Food and Drug Administration (FDA) [21, 22], there is no cure for DMD; however, proper approaches could slow down the nature course of the disease, improve longevity and quality of patients’ life [23]. Therefore, genetic testing offers a comprehensive and conclusive means of identifying mutations in the dystrophin gene or other relevant genes, guiding clinicians toward an accurate diagnosis and early intervention.

Considering most of DMD patients were resulted from large deletion and/or duplications in the DMD gene, the standard or recommended genetic testing for patients with DMD-like symptoms would be quantitative analysis to detect deletions and/or duplications of DMD exons by MLPA or array-cGH, followed by WES or Sanger seuqencing and if necessary, whole genome sequencing (WGS) when MLPA or array-cGH returns negative results [24]. The fast development of WES brings higher accuracy in determining copy number variations of exons, as well as lower cost, WES would be used as first choice for further diagnosis for DMD-like symptoms. For females with DMD-like symptoms, though the recommended protocol was used, we would suggest a WES-first protocol for diagnosis in the future.

In summary, we have identified a novel variant in the *DMD* gene within a large DMD family, where a carrier exhibits DMD-related symptoms due to non-random X-chromosome inactivation. Our finding contributes to the broadening of the spectrum of DMD mutations. The integration of gene testing with clinical phenotype assessment can be a valuable tool for physicians in the diagnosis of progressive muscular dystrophy, particularly for females presenting with DMD-like symptoms.

### Electronic supplementary material

Below is the link to the electronic supplementary material.

Supplementary Figures

## Data Availability

The datasets generated for this study can be found in the NCBI Sequence Read Archive (SRA) database (accession number: PRJNA1031384), which will be available upon request.
