# Supplementary material for: A female patient carrying a novel DMD mutation with non-random X-chromosome inactivation from a DMD family
Source: BMC Med Genomics. 2024 Feb 1;17:46. doi: 10.1186/s12920-024-01794-x (PMC10832127; doi:10.1186/s12920-024-01794-x)

**A female patient carrying a novel *DMD* mutation with non-random X-chromosome inactivation from a DMD family .**

Ming-Xia Sun^1,#^, Miao Jing^1,#^, Ying Hua^1*^, Jian-Biao Wang^1^, Sheng-Quan Wang^1^, Li-Lan Chen^1^, Liang Ju^2^, Yan-Shan Liu^3*^

1. Department of Neurology, Affiliated Children's Hospital of Jiangnan University (Wuxi Children's

Hospital), Wuxi, China

1. Department of Cardiology, Affiliated Children's Hospital of Jiangnan University (Wuxi Children's Hospital), Wuxi, China
2. Department of Pediatric Laboratory, Affiliated Children's Hospital of Jiangnan University (Wuxi Children's Hospital), Wuxi, China

#These authors contributed equally to this study.

*Corresponding authors:

Ying Hua, Department of Neurology, Affiliated Children's Hospital of Jiangnan University (Wuxi Children's Hospital). E-mail address: [tomato331316@163.com](mailto:tomato331316@163.com)

Yan-Shan Liu, Department of Pediatric Laboratory, Affiliated Children's Hospital of Jiangnan University (Wuxi Children's Hospital). E-mail address: Liu_yshan@hotmail.com

Supplementary Figure legends.

Supp Figure 1. Sanger sequencing results of other family members.

Supp Figure 2. Non-random X-chromosome inactivation testing result of the twin sister (III-1).


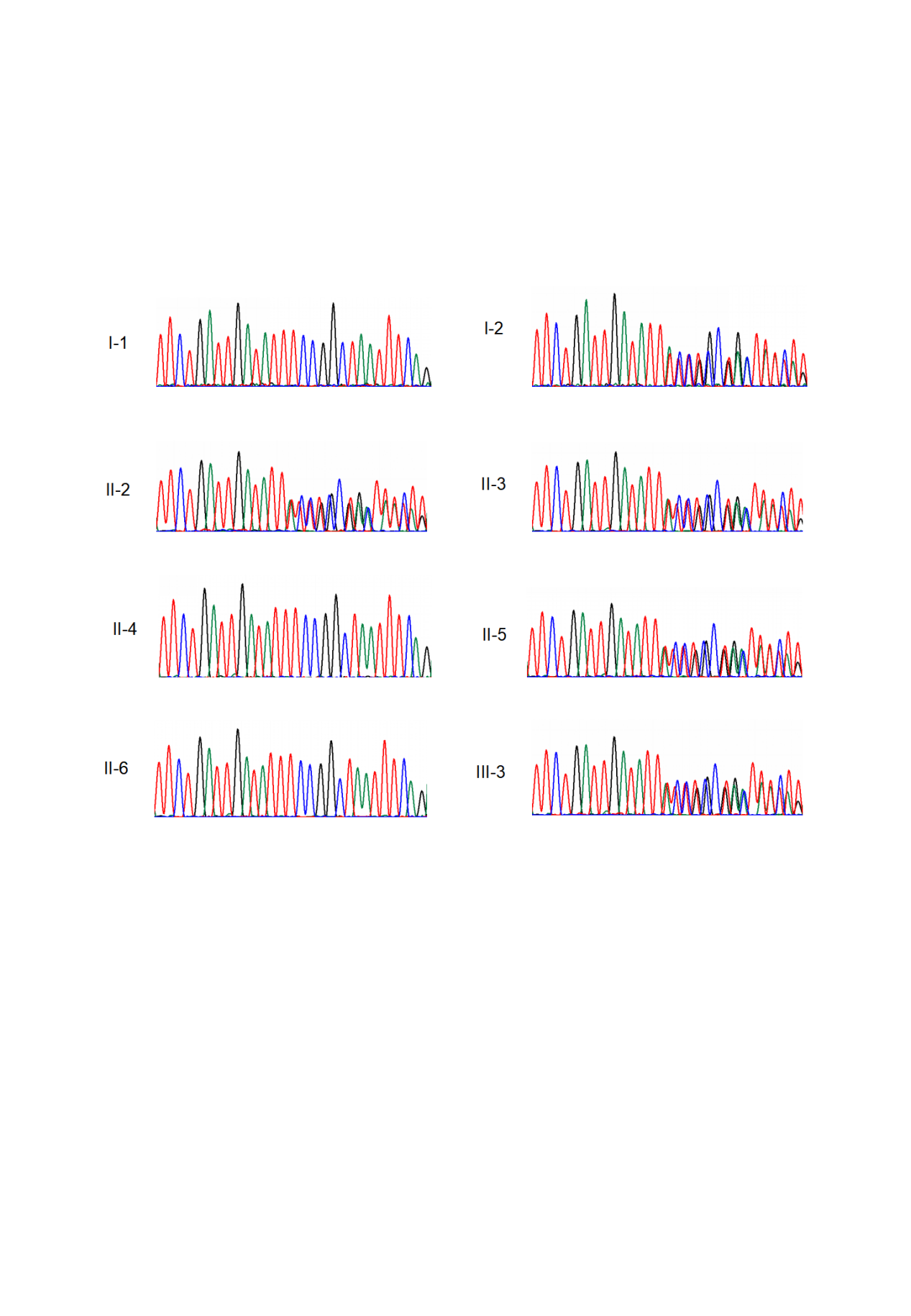


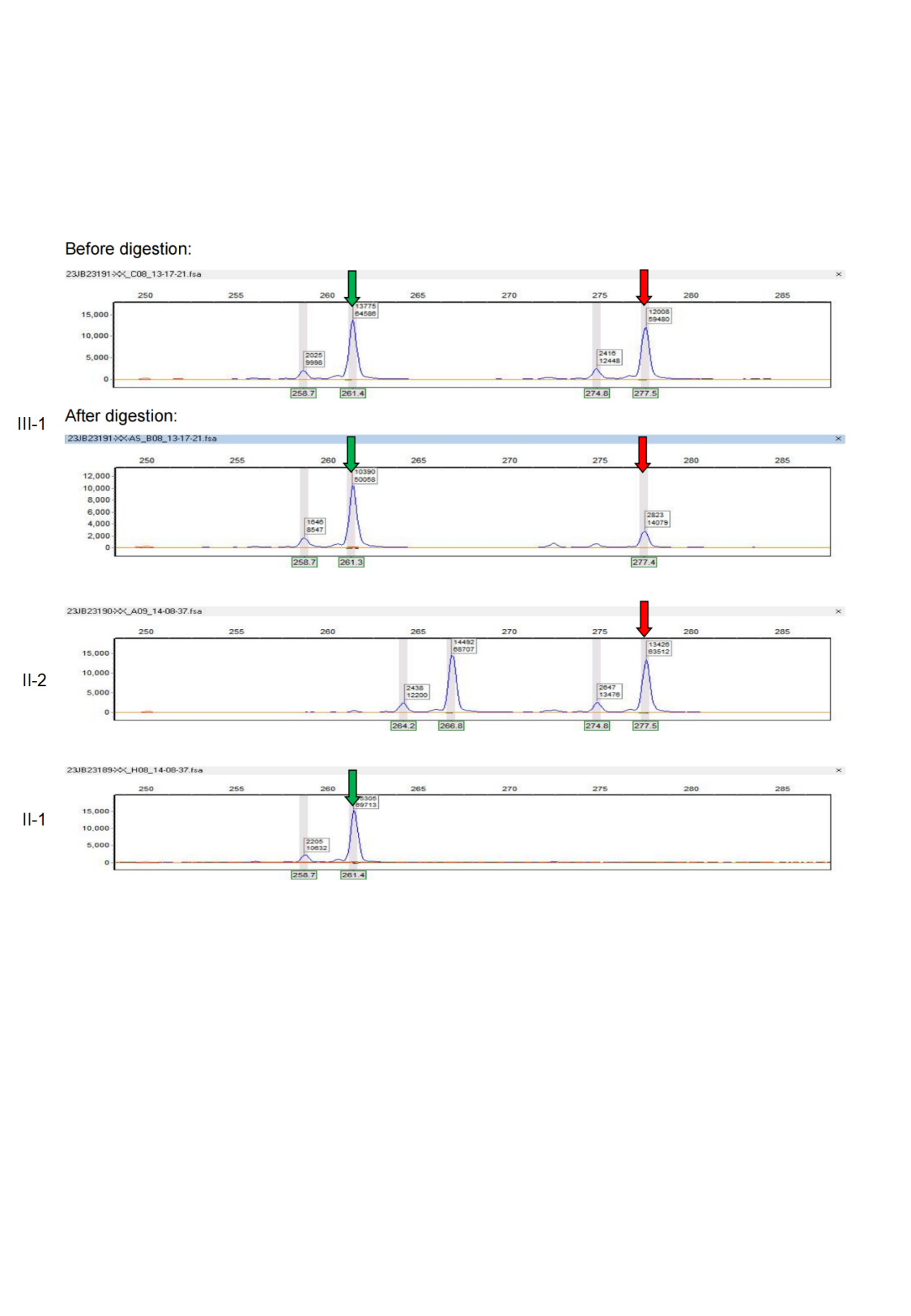

Supplement: Supplementary file 1 — Supplementary Figures [file 12920_2024_1794_MOESM1_ESM.docx]
